# Supplementary material for: A metagenomics survey of viral diversity in mosquito vectors allows the first detection of Sindbis virus in Burkina Faso
Source: PLoS One. 2025 Jun 12;20(6):e0323767. doi: 10.1371/journal.pone.0323767 (PMC12161561; doi:10.1371/journal.pone.0323767)

Supplementary information

S2 Table. Primers used to amplify and sequence gaps in the SINV genome.

Amplicon	Primer couple	Forward primer	Reverse primer	Amplicon length
1	Sind121F/Sind907R	CCAAATGACCATGCTAATGCC	TGATCCCGGGACTGATGGTG	787
2	Sind1537F/Sind2055R	CTCCGAGAAGCACTCCCA	TCGCTTCTTGTCCACGTC	519
3	Sind3793F/Sind4719R	GCCCTGAATTGCCTTAACCC	GAACAGGACCTTTATCTCCGCC	927
4	Sind6841F/Sind7604R	TCGTTCGACAAAAGCCAAGAC	CATGGTGGTGGTGTTGTGGT	765
5	Sind8719F/Sind9589R	TTCAGACTTCCGCCCAGTTC	GGTTCATGATTGCCCCATATG	871
6	Sind9661F/Sind10962R	ACTACCACCGCCACCCTGTG	TCCCGCCGAAGTCTGCTGAG	1 302

S1 Script. R code used to estimate the SINV infection rate.

```
library(binGroup)

x1 <- c(1, 0) # number of positive pools per pool size

m1 <- c(22, 30) # pool sizes

n1 <- c(1, 5) # number of pools per pool size

pooledBin(x=x1, m=m1, n=n1, scale=100)
```

S3 Table. Metadata of the pools from the Rural 1 zone obtained in 2020, including information on the pool that led to SINV isolation in cell culture (SINV_positive = 1).

pool	species	site	year	mosquitoes	library	SINV_positive
Cx_R1_20_2_1	Cx_quinquefasciatus	Rural1	2020	22	Cx_R1_20_F_2	1
Cx_R1_20_2_2	Cx_quinquefasciatus	Rural1	2020	30	Cx_R1_20_F _2	0
Cx_R1_20_3_1	Cx_quinquefasciatus	Rural1	2020	30	Cx_R1_20_F _3	0
Cx_R1_20_3_2	Cx_quinquefasciatus	Rural1	2020	30	Cx_R1_20_F _3	0
Cx_R1_20_4_1	Cx_quinquefasciatus	Rural1	2020	30	Cx_R1_20_F _4	0
Cx_R1_20_4_2	Cx_quinquefasciatus	Rural1	2020	30	Cx_R1_20_F _4	0

S1 Fig. Maximum-likelihood phylogeny based on the full ORF sequence of the E2 gene. The sequence from Burkina Faso is indicated with a black square. The scale bar represents the number of nucleotide substitutions per site. GenBank accession numbers are indicated on the branch names. The phylogenetic tree was generated following the procedure described in the Materials and Methods section of the main text.

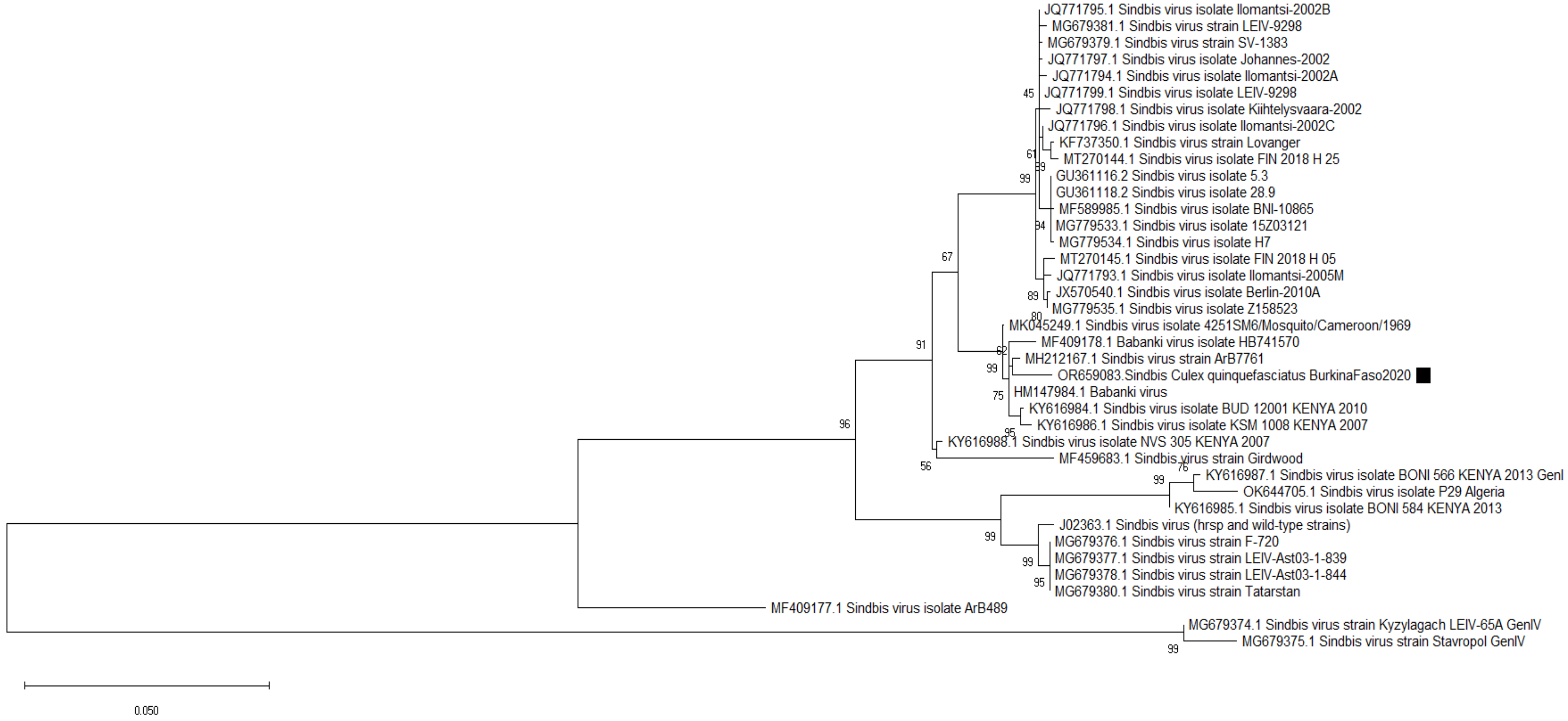

Supplement: S1 Data — This file contains S2 Table, S1 Script, S3 Table and S1 Fig. The titles of each item are: S2 Table. Primers used to amplify and sequence gaps in the SINV genome. S1 Script. R code used to estimate the SINV infection rate. S3 Table. Metadata of the pools from the Rural 1 zone obtained in 2020, including information on the pool that led to SINV isolation in cell culture (SINV_positive = 1). S1 Fig. Maximum-likelihood phylogeny based on the full ORF sequence of the E2 gene. The sequence from Burkina Faso is indicated with a black square. The scale bar represents the number of nucleotide substitutions per site. GenBank accession numbers are indicated on the branch names. The phylogenetic tree was generated following the procedure described in the Materials and Methods section of the main text. (PDF) [file pone.0323767.s002.pdf]
