# Supplementary material for: A metagenomics survey of viral diversity in mosquito vectors allows the first detection of Sindbis virus in Burkina Faso
Source: PLoS One. 2025 Jun 12;20(6):e0323767. doi: 10.1371/journal.pone.0323767 (PMC12161561; doi:10.1371/journal.pone.0323767)
Supplement: Attachment — Submitted filename: reviewers_comments_PNTD_v3.docx [file pone.0323767.s003.docx]

Dear editor and reviewers,

Please find below the point-by-point answers to the reviewers’ comments. Answers are always in Calibri font to facilitate visualization. We thank the reviewers for the significant improvement of the manuscript.

We are very sorry for the delay in providing our answer. We have not been able to obtain the license for the shapefile for Figure 1 from the Burkinabe public body that provided it. PLoS requires the license terms. The absence of answer from the IGBK explains most of the delay.

Sincerely,

Serafín Gutiérrez

***********************

Reviewer's Responses to Questions

Key Review Criteria Required for Acceptance?

As you describe the new analyses required for acceptance, please consider the following:

Methods

-Are the objectives of the study clearly articulated with a clear testable hypothesis stated?

-Is the study design appropriate to address the stated objectives?

-Is the population clearly described and appropriate for the hypothesis being tested?

-Is the sample size sufficient to ensure adequate power to address the hypothesis being tested?

-Were correct statistical analysis used to support conclusions?

-Are there concerns about ethical or regulatory requirements being met?

Reviewer #1: The object of the study is clear and the study design is well defined. The material is a valid sample for surveillance and the methods suit the study design well. The material covers several years, two important mosquito vector species and both rural and urban areas.

Reviewer #2: In general, the methods in this manuscript are described well. However, I do have some questions regarding the mosquito pooling and subsequent processing before sequencing, the bioinformatic processing of the reads, and the mosquito infection rate estimation based on the qPCR.

1) 60 and 47 pools were created for Cx. quinquefasciatus and Ae. aegypti, respectively and mosquito numbers per location and year are shown in Table 1. However, I wasn't able to find the number of mosquitoes per pool, in Table 1 only the total number of mosquitoes per total number of pools is shown. Was there a specific target number of mosquitoes per pool? I would like to see a bit more information on this. In addition, it was not clear to me throughout the manuscript that some pools were pooled together into one library for sequencing. Also here information on which pools were pooled together is lacking.

Whenever possible, we set the number of mosquitoes per pool to 30. This number had to be adapted depending on catch size. Thus, pool size varied between 6 and 37 individuals, with a median of 30. This information is now provided in the main text (lines 158-160). In addition, information on the pools, the number of individuals per pool, and the library associated to each pool is now provided in Table S1. Finally, we have identified a typing error in the number of pools from Urban 1 zone (13 pools in 2020 instead of 14). This error does not change total mosquito number or our main results. The text and Table 1 have been modified to correct for this error. We apologize for the error.

2) On line 168, the authors mention that they used the method of reference 34 (Temmam et al., 2015) and shortly describe their procedure. However, in reference 34 a viral purification by filtration is used (0.45µm filter), which is not mentioned in the short description. Did the authors also use filtration for viral purification (highly recommended in my opinion)? If so, they should mention this and provide the filter size.

There is an error in the citation. We thank the reviewer for having spotted this error. The correct citation is a previous paper from our group (Gil et al., 2021). We have amended the text accordingly. The correct citation analyses, among others, the efficiency of the filtration step described in the Temmam’s paper. The filtration step led to a limited reduction in non-viral nucleic acids. Moreover, filtration led to a loss of sample volume. These observations led to the removal of the filtration step from our protocol.

On line 191, "AmpurXP" should be "AMPure XP", additionally I would like to see in which ratio (beads to sample) they used the beads. This is important as it will have an influence on the size selection of the DNA fragments being purified.

The kit name has been corrected and the bead ratio (0.6) is now provided in line 197.

3) On lines 203-205, the authors mention that the reads from all libraries were pooled together and used for one de-novo assembly, which is also called a cross-assembly. I would generally advice against performing cross-assemblies as they can create non-existing genomes or chimera's. If the authors do not have a specific reason to do a cross-assembly, I would assemble all samples separately (less computationally intensive!) and cluster the resulting contigs of all samples subsequently to create the non-redundant set of contigs.

We agree that cross assembly can lead to chimeras. However, this approach can, under appropriate parametrization, improve the recovery of viral genomes from sequencing data obtained with shotgun metagenomics from mosquito samples. Generating viral contigs is a challenge due to the limited number of viral reads that is usually obtained with shotgun metagenomics. In our hands, cross assembly alleviates this problem and improves the assembly of viral contigs. This positive effect could be due to an increase in the information injected in the De Bruijn graphs (i.e., more viral reads). In addition, we check for chimeras in viral contigs (*e.g*., blast output with several gaps) and we do not often find chimeras. Moreover, the problem of computational resources is largely alleviated with the use of MEGAHIT, an assembler specifically designed for speed and memory efficiency (Li et al., 2015). Finally, the cross assembly used in the paper has been published before (e.g., Gil et al., 2021 Mol. Ecol. Res., Morel et al., 2024 PloS One). Hence, we would like to keep the cross-assembly method unless demanded by the reviewer.

4) The Sanger sequencing and qPCR is by far the least explained part of the Methods. Certainly information on how the infection rate in the mosquitoes is estimated, is not described and only the name of an R package is given. I would like to see a more in-depth explanation of how the authors came to a result of 1.25% infection rate. In addition, also the R code and underlying data should be made available for readers to understand how everything was calculated.

We apologize if the description of these methods was not precise enough. The current version provides a description of the method applied in the BinGroup package (lines 224-233, see text below). Briefly, the estimation is based on a maximum likelihood approach that estimates infection rates from pools with different pool sizes. The maximum-likelihood estimation is considered the more appropriate method for infection rate estimation (Gu et al., 2003 *J Med Ento*). As requested, the data and the R code are now provided to readers as supplementary information.

The infection rate was estimated from the six pools obtained in the Rural 1 zone in 2020, the sampling that provided a SINV-positive library and a cell culture infection. Those pools included one pool with 22 individuals and five pools with 30 individuals (172 females in total). Two pools were considered PCR-positive for SINV RNA (see table below with Ct values). The Ct values are now provided in the manuscript (line 292). Analysis of these data with the pooledBin function in the BinGroup package provides an estimate of 1.25% (CI = 0.24/4.26). The current manuscript version only considers one pool as SINV positive. This change follows reviewers’ suggestions below. The only pool considered SINV-positive was the one that led to an isolation in cell culture. The analysis of the updated dataset provides an infection rate with confidence intervals that overlap those of the previous estimate (infection rate = 0.57%; CI = 0.03/2.82). The new infection rate is also similar to those found in mosquito populations from outbreak sites in Northern Europe, a point that is part of the Discussion section. In addition, we have modified the Discussion to take into account the fact that the current infection rate is lower than the previous one (lines 361-367).

Text added in the Materials and methods section: “This package provides functions to estimate the infection rate from the analysis of pools or groups, rather than individuals, for virus infection. Here, estimates and their confidence intervals were obtained using the pooledBin function. This function follows a maximum likelihood approach to calculate confidence intervals for a single infection rate based on tests of pools containing pools of different sizes. We estimated the infection rate in the Rural 1 zone in 2020, the only zone and year in which a SINV-positive library was detected. The dataset and the R script are available in the Supplementary Information 1 file. Only the pool that resulted in SINV infection in cell culture was considered as SINV positive”.

Table. Dataset used in the previous version to estimate infection rates (provided as Table S3 in the manuscript). The current version uses an updated version of the dataset as described above.

| pool | library | replicate | species | zone | year | females/pool | PCR_positive | Ct value |
| --- | --- | --- | --- | --- | --- | --- | --- | --- |
| Cx_R1_20_2_1 | Cx_R1_20_2 | 1 | Culex | Rural1 | 2020 | 22 | 1 | 22 |
| Cx_R1_20_2_2 | Cx_R1_20_2 | 2 | Culex | Rural1 | 2020 | 30 | 1 | 33 |
| Cx_R1_20_3_1 | Cx_R1_20_3 | 1 | Culex | Rural1 | 2020 | 30 | 0 | >40 |
| Cx_R1_20_3_2 | Cx_R1_20_3 | 2 | Culex | Rural1 | 2020 | 30 | 0 | >40 |
| Cx_R1_20_4_1 | Cx_R1_20_4 | 1 | Culex | Rural1 | 2020 | 30 | 0 | >40 |
| Cx_R1_20_4_2 | Cx_R1_20_4 | 2 | Culex | Rural1 | 2020 | 30 | 0 | >40 |

--------------------

Results

-Does the analysis presented match the analysis plan?

-Are the results clearly and completely presented?

-Are the figures (Tables, Images) of sufficient quality for clarity?

Reviewer #1: The aim of the study was to look into metagenomics of the virus diversity of important vector mosquito species in Burkina Faso. The authors' have conducted metagenomic analyses for a decent dataset (~3000 mosquito sample). The authors' discovered 103 viruses, out of which 87-88 were specific for arthropods. Yet, the only listed virus here is Sindbis virus (SINV) and these other viruses are not listed in the study which species etc. (not in the main text, supplementary material or submitted to GenBank). This raises a question that why to conduct a metagenomic analyses if not analysing it further? In my view, a significant portion of the data appears to be underutilized or not effectively leveraged.

1) The lack of data might indicate that there were no other significant findings in authors' opinion in this material. Yet, if the aim is to study the virus diversity, reporting presence of SINV does not meet with this aim.

We apologize for not having described in detail the reasons behind the lack of data on other viruses in our dataset. There is a second manuscript associated to this work in which the virus diversity is described in detail, including GenBank accessions. In fact, we had two goals when we conducted the study. The first goal, the goal of this manuscript, was to explore arbovirus diversity in main mosquito vectors. We only detected one arbovirus: SINV. The second goal was to analyze the influence of mosquito sex on the virome. To this end, we not only collected females but also males, in similar numbers, and we have compared their viromes. Thus, the second manuscript, the one describing in detail the viral diversity, focuses on a subject that is not directly related to arbovirus prevalence. This situation explains why we chose to separate the results of the two goals. We have included a sentence in the manuscript explaining the availability of data on the full virus diversity in another manuscript (Lines 264-265; “Since the goal of this work is to detect arboviruses, the diversity of mosquito-specific viruses is described in a companion manuscript (under preparation)”.

Reviewer #2:

1) A general comment I have is that the Results section feels rather short, probably because it is presented as a separate manuscript from other analyses that were performed on the same dataset (virome exploration, not published?, and arbovirus screening by PCR described in references 20 and 21). This could be mitigated by adding some extra phylogeograpical analyses with the detected SINV genome (similar to reference 16 [Ling et al., 2019] or <https://doi.org/10.1038/s41467-023-42185-7>). This could also further substantiate the authors' claim that West Africa should also be considered as a reservoir of SINV that spills into Europe.

As guessed by reviewer #2, there is a companion manuscript under preparation describing the full diversity of mosquito-specific viruses. The arguments explaining the two manuscripts are provided right above this comment, in the answer to a similar comment by reviewer #1.

The idea of generating a detailed phylogeographical analysis is appealing. We have discussed on this point with experts in phylogeography using Bayesian inference, the method required for a robust analysis of the question (colleagues from Sebastien Duchene’s lab, Pasteur Institute). Their opinion is that our dataset is not adapted to a robust phylogeography analysis because we only have one sequence from Western Africa. For example, the article proposed as example by reviewer #2 generated 19 European sequences and seven African sequences. Together with the sequences in public databases, they included more than 500 sequences to robustly explore virus phylogeography. Hence, for the sake of conciseness, we prefer not to add extra analyses that are not going to improve the phylogenetic analysis already provided in our manuscript.

Given the limits in our sequence dataset, our previous version already described the role of West Africa as the source of European SINV strains as “potential” (line 316 in previous version). Having said that, we acknowledge that other scenarios were not explicitly discussed. For example, West Africa could be a recipient of strains from Central Africa, without being involved in SINV dispersal into Europe. The current version now describes other possible scenarios in the Discussion (lines 334-338; the new text: “Thus, West Africa should also be considered among the potential geographic sources of the European strains. However, other scenarios could have taken place. For example, West Africa could simply be a recipient of strains from Central Africa, without being involved in the spread of SINV to Europe. More sequences from West Africa are needed to address this question through a robust phylodynamic analysis”).

2) Furthermore, I have some major concerns on the infection rate calculation. On lines 273-274, the authors mention that 2 pools were found positive for SINV, but they both belonged to the same sequencing library. It would be an enormous coincidence that out of the 66 libraries screened (107 pools), just the 2 pools positive for SINV would be used together for one library (from Rural zone 1 in 2020 there were 6 pools available and those were eventually pooled into 3 libraries). Together with the fact that the authors were not able to isolate SINV from one of the pools, this suggests to me that there was contamination (probably during the extraction). This has of course implications for the downstream infection rate calculation which is based on the qPCR results. I recommend to re-extract the homogenate of the pool with the lowest concentration and perform the qPCR again on this new extract. I also would like to see some Ct values (or genome copy numbers) for these positive samples together with more information how you calculate the maximum-likelihood estimate of the infection rate from the qPCR results.

We agree with reviewer #2 on that we cannot rule out a potential contamination. Unluckily, the homogenates of the PCR-positive pools were used for cell culture inoculation. We cannot re-extract and test again the pools. Therefore, we have modified our infection rate calculation to avoid a potential overestimation due to a contamination. Only the pool that led to an isolation, a solid proof of virus presence, is now considered as SINV positive for the calculation of the infection rate (for a detailed explanation see the answer to comment 4 of the Methods section by reviewer #2). Moreover, the Ct values are now provided in the main text (line 294). Finally, as described in the answer to comment 4 of the Methods section by reviewer #2, the Methods section now includes a detailed explanation of the infection rate estimation, including the dataset and the R code (lines 224-233; Supplementary Information 1).

Other minor comments:

- lines 240-242: I'm not able to calculate the median number of mosquitoes per library from Table 1.

The median number per library is 30 mosquitoes. We have included Table S1, a table with more details on the pools and libraries to allow to estimate the median, among other statistics.

- line 244-245: I think the numbers here should be "107 million" and "109 million" instead of "10.7 million" and "10.9 million".

We thank the reviewer for having spotted an error. The numbers are correct but the variable is not. The numbers refer to the number of viral reads and not to “total reads”. The text has been corrected (lines 261-262).

Conclusions

-Are the conclusions supported by the data presented?

-Are the limitations of analysis clearly described?

-Do the authors discuss how these data can be helpful to advance our understanding of the topic under study?

-Is public health relevance addressed?

Reviewer #1:

1) Conclusions are on line with the results except the conclusion on the infection rate. In my opinion this is over interpreting as with these numbers this estimation should not be done. My main criticism is about the dataset and the methods allowing for much wider analyses than is presented in the study.

The infection rate estimation was based on the analysis of six pools including 172 mosquitoes in total. This dataset is big enough to infer the infection rate. It should be noted that 95% confidence intervals (CI) were provided. The width of the CI range allows to determine the estimate precision. Here, the current CI are 0.03% and 2.82%, thus allowing readers to predict that the infection rate is probably rather low. Thus, we think these results are of interest to the community and should be part of the manuscript.

Having said that, we agree that the limited extent in number, time period and geographical range of SINV-positive pools was low to draw robust conclusions on SINV prevalence. The previous version already acknowledged this issue in lines 326- 328 (“In this study, the limited number of *Cx. quinquefasciatus* mosquitoes per year and zone (116 females on average) hampered a robust quantitative analysis of SINV prevalence over years.”). We have modified the manuscript to further underline the limits of the infection rate estimate. First, we have included in the discussion a statement on the limits of our results (lines 363-367; the new text: “However, the limited extent of the number, geographic range and time period of SINV-positive pools did not allow for an accurate estimate, as shown by the confidence intervals. This situation does not allow robust conclusions to be drawn on SINV prevalence. Larger mosquito sampling and screening campaigns are needed to accurately assess the prevalence of SINV in mosquitoes in the region”). Moreover, the statement on the potential for spillover due to the observed infection rate has been removed from the discussion (lines 344-345 in the previous version).

Reviewer #2:

1) The authors clearly describe the advantages and disadvantages of metagenomics in the context of arbovirus surveillance and how it could benefit public health. They also mention the weaknesses of their study related to the limited reporting of SINV infection in (West) Africa. My main concerns on their conclusions are based towards the SINV prevalence in mosquitoes and the potential spillover risk (lines 339-342) as I suspect these results might be a result of contamination.

We understand the concern of reviewer #2 and this is why we have recalculated the infection rate. The new estimation does not use PCR data; it is only based on data from the most robust detection method, the isolation in cell culture. This choice drastically reduces the chances that contamination influenced our results. These changes are described in detail in the answers to previous comments of the same reviewer (see answers to comment 4 in the Methods section and to comments in the Results section). Moreover, we have also modified the discussion to lower claims on the potential consequences of the infection rate estimate. Briefly, we have explicitly stated the width of the confidence intervals and its impact on estimate precision. We have also removed the statement on the potential link with a spillover. Please, see right above the answer to the comment of reviewer #1 for more details on these changes in the discussion.

--------------------

Editorial and Data Presentation Modifications?*<br/*>

Use this section for editorial suggestions as well as relatively minor modifications of existing data that would enhance clarity. If the only modifications needed are minor and/or editorial, you may wish to recommend “Minor Revision” or “Accept”.

Reviewer #1: The manuscript would benefit a lot from thorough language checking and corrections. Another improvement for the manuscript would be adding consistency. E.g. use either 1 000 with a space as in line 224 or without space as in 1629 and 1356 in line 159. Consistency is needed with the terms used. Be specific are you discussing mosquito-borne viruses or arboviruses. Finally, the use of abbreviations is lacking totally. Please use the abbreviations. Indicate the abbreviation first time a term is used and subsequently use the abbreviation. The detailed comments are listed in a separate file.

We thank reviewer #1 for the in-depth manuscript edition and the manuscript improvement. All comments have been reviewed and modified. Moreover, the manuscript has gone through language checking. Please, see below the point-by-point answers.

- Specific comments on the manuscript:

Introduction:

- Line 66-68: The sentences. “Moreover, this diversity includes a variety of life cycles, differing in the species of mosquito vectors and vertebrate hosts [2]. Not surprisingly, different epidemiological situations are associated to these viruses.” should be modify to improve clarity of the message.

The new version is “This diversity includes viruses with different life cycles (*e.g*., differing in the mosquito and vertebrate species they infect) [2]. Not surprisingly, these viruses are associated with different epidemiological situations”.

- Line 68: add (DENV) after dengue virus and use the abbreviation afterwards.

Abbreviation of virus names have been added to those virus names appearing more than once in the text.

- Line 72: Authors state “Prevention and control of arboviruses therefore mainly rely on surveillance.” Please add the vector control here as it is in crucial trole in prevention of arboviral diseases.

The new version is: “Prevention and control of arboviruses therefore rely mainly on surveillance and vector control“ (lines 71-72).

- Line 74: Please clarify what you refer to with “among others”

“Among others” here refers to, for example, to alert doctors on virus circulation and thus improve diagnosis. For the sake of conciseness, the term “among others” has been removed.

- Lines 79-80: Please add (MBV) after mosquito-borne viruses and use the abbreviation afterwards.

This comment is related to a previous comment by reviewer #1 (in the main comments of the Editorial and Data Presentation Modifications). The previous comment stated that more specificity was required when using the terms mosquito-borne virus and arbovirus. In the previous version, these terms were used to distinguish between all arboviruses (transmitted by mosquitoes or other arthropods) and arboviruses transmitted by mosquitoes. However, the term mosquito-borne viruses may be misleading as mosquito-specific viruses (i.e., viruses that do not infect vertebrates; a term also used in the manuscript) can also be considered as mosquito-borne viruses. To improve precision, the term “mosquito-borne viruses” has been replaced by “arboviruses” or “mosquito-borne arboviruses” in the manuscript.

- Lines 92-93: Wouldn’t screening of the antibodies from host animals in some cases perhaps be more efficient than screening viruses from mosquitoes to survey which pathogens are around?

Several zoonotic arboviruses, including SINV or West Nile virus, infect wild vertebrates. Sampling wild vertebrates is usually difficult due to both technical and legal constraints. Mosquito surveys allow to overcome those constraints. Having said that, serosurveys of infections in humans by those viruses can be an efficient alternative to surveys of mosquito populations. Serosurveys in humans are now proposed in the manuscript (lines 321-323).

- Lines 97-98: Please state which MBVs, at least the ones with most effect on public health.

The new sentence is “Several arboviruses, including DENV, Zika virus (ZIKV), chikungunya virus (CHIKV), West Nile virus (WNV), Yellow fever virus and Rift Valley fever virus, have been previously detected in Burkina Faso” (lines 97-98).

- Lines 105-106: Please provide abbreviations for the viruses which are mentioned latter in the text.

Done.

- Lines 115-120: Please be consistent. Do the outbreaks occur only in Northern Europe or in Northern Europe (and Russia) and in Africa.

To improve precision, countries with periodic outbreaks are now indicated (“To date, periodic outbreaks of SINV infections have been reported only in Finland, Sweden, Russia and South Africa [27,30].” (lines 115-117).

Materials and methods

- Figure 1. Please provide the map (inner panel) zoomed out with borders and names of neighboring countries to provide clearer geographical content.

A new version of Figure 1 is now provided (lines 148-152). This version includes the improvements suggested by Reviewer 1.

- Line 153: Details (e.g., manufacturer) are lacking for the traps. Secondly please replace aspiration with aspirator.

Done.

- Line 155: Please provide information on if the mosquitoes were stored as dry or with media? If with media, which one? This is extremely important to be explained as it crucially affects virus viability.

Mosquitoes were stored without media to avoid potential interference with the enzymatic reactions required for library preparation. The text now contains the storage conditions (i.e., “dry”; lines 162-163).

- Line 158: Please specify how many mosquitoes/ pool. Also, the mosquitoes were probably pooled by the species also?

A new paragraph with the information on pooling has been generated (lines 160-166; text provided below). Moreover, a new table, Table S1, provides detailed information on the pools (e.g., pool size and pool/library associations). The new text is: “Immediately after species identification, non-blood-engorged females were pooled by mosquito species, date and collection zone (Table 1). Pool size was set at 30 individuals but had to be adjusted depending on catch size (median/minimum/maximum pool size = 30/6/37; Table S1). Pools were immediately stored dry at -80℃ for subsequent analyses”.

- Line 206: Specify the version of CAP3

There is only a single version of CAP3, the one identified in the citation in the manuscript.

- Lines 220-224: Define the root of the tree. Did the authors use the whole genomes or only ORFs? If ORFs were used, was it whole or partial ORFs for the trees? How and in which order the ORFs were combined for the alignment or was the combining done after alignment?

The root was defined using two sequences from genotype VI. This information is now provided in lines 285-287.

The sequences were concatenated ORFs (NS and S ORFs; now indicated in the figure legend, line 281, and in the Methods section, line 236). That is, the sequences did not contain the non-coding regions. The concatenation followed the SINV genome structure (first NS and then S ORFs). The concatenation was done before the alignment.

- Line 228: Please add “and incubated at 37°C”

Done.

- Line 233: If tested only at one timepoint how do authors exclude the possibility of carryover?

We excluded a possible carryover because the Ct values in culture supernatant were much lower than that in the inoculum (22 versus 7; this information is now provided in line 298). The difference in Ct values implies that the concentration of SINV nucleic acids had increased several logs compared to that in the inoculum.

Results

- Line 263:- Please refer in figure legend for figure 2 to the previous study with the Algerian Sindbis sequence.

We have added a citation to the study.

- Line 276: Please replace cytopathological with cytopathic

Done.

- Line 277: Please provide the results of the qPCR

The Ct value is now provided (Ct 7; line 298).

- Lines 288-290: Please provide the most important resulst of the previous study.

The mosquito screening for arboviruses in previous studies did not detect any of the targeted arboviruses. This information was already provided in lines 290-292 (currently, lines 310-312).

- Line 300: Please rephrase the possible impact on human health

Previously: “Future studies on the epidemiology of SINV infections in Burkina Faso are needed to assess its potential impact on human health.”

Current version (lines 321-322): “Studies on the prevalence (*e.g*., serosurveys) and symptomatology of SINV infections in humans are needed to assess the potential health impact of SINV in Burkina Faso.”

- Line 324: What is known about wild birds in the study area? Are there bird species which have been studied being involved in SINV transmission cycle?

There is a large diversity of birds in the study area ([Boano](https://sciencepress.mnhn.fr/sites/default/files/articles/pdf/zoosystema2022v44a2.pdf) et al., Zoosystema 2022). However, the information on SINV-infections in wild birds in Africa is scarce. Most studies on the avian host range of SINV come from European settings. SINV antibodies have been detected in several migratory bird species in Europe and also found in Burkina Faso (*e.g*., *Lanius collurio, Sylvia atricapilla, Sylvia borin, Sylvia communis, Phylloscopus trochilus*) (Kurkela et al., 2008, *Emerging Infectious Diseases*; Hesson et al. 2016, *PLoS One*).

Reviewer #2: I think the manuscript would benefit from thorough copyediting before publication, a few suggestions:

We thank Reviewer #2 for the in-depth manuscript edition and the manuscript improvement. All comments have been reviewed and modified. Moreover, the manuscript has gone through language checking. Please, see below the point-by-point answers.

line 54: felt -> fell

Done.

line 68: associated to -> associated with; case number -> case count

Done.

line 70: case number -> case count

Done.

line 76: tenths of -> dozen/tens of

Done.

line 77: renders complex -> complicates

Done.

line 78: among others -> primarily

Done.

line 83: the latter -> these viruses

Done.

line 127: routes of spread -> dissemination routes

Done.

line 217: defined -> designed?

Done.

line 258: felt -> fell

Done.

line 290: ..., just like found here in the metagenomics survey. -> which we confirmed with this metagenomic survey.

Done.

line 312: felt -> fell

Done.

line 318: felt -> fell

Done.

line 320: incriminated -> investigated?

Incriminated has been replaced by involved or identified.

--------------------

Summary and General Comments

Use this section to provide overall comments, discuss strengths/weaknesses of the study, novelty, significance, general execution and scholarship. You may also include additional comments for the author, including concerns about dual publication, research ethics, or publication ethics. If requesting major revision, please articulate the new experiments that are needed.

Reviewer #1: Kaboé et al provide a manuscript on "A metagenomics-based survey of the virus diversity in mosquito vectors allows the first detection of Sindbis virus in Burkina Faso". I think the topic is relevant as it is well known that the changes in the environment do affect the seasonality, the distribution and the emergence of mosquito-borne diseases. This kind of surveillance is crucial in outbreak preparedness and also in mitigation to environmental change.

1/ Despite the relevant topic, the manuscript does not cover the study on virus diversity as it is only focused on Sindbis virus, leaving all the rest of the data unanalysed. The authors even state that it is out of the scope of this study. I strongly disagree with this. This would very much be relevant data about the current situation and reference material for the future. I am suggesting adding at least highlights on the rest of the data (at least on the viruses capable of infecting vertebrates).

This comment is a follow-up of a similar comment by reviewer #1 in the Results section. The answer to the comment is again provided below to facilitate review of our answers.

We apologize for not having described in detail the reasons behind the lack of data on other viruses in our dataset. There is a second manuscript associated to this work in which the virus diversity is described in detail, including GenBank accessions. In fact, we had two goals when we conducted the study. The first goal, the goal of this manuscript, was to explore arbovirus diversity in main mosquito vectors. We only detected one arbovirus: SINV. The second goal was to analyze the influence of mosquito sex on the virome. To this end, we not only collected females but also males, in similar numbers, and we have compared their viromes. Thus, the second manuscript, the one describing in detail the viral diversity, focuses on a subject that is not directly related to arbovirus prevalence. This situation explains why we chose to separate the results of the two goals. We have included a sentence in the manuscript explaining the availability of data on the full virus diversity in another manuscript (Lines 264-265; “Since the goal of this work is to detect arboviruses, the diversity of mosquito-specific viruses is described in a companion manuscript (under preparation)”.

2/ I would also remove the analyses on the infection rate as with these numbers the analyses is irrelevant.

*Both reviewers have indicated issues concerning the infection rate analysis (e.g., comments 1/Conclusions by reviewer #1; 4/Methods and 2/Results by reviewer #2).*

Estimates of the infection rate are needed to asses infection risk and to analyze potential changes in arbovirus prevalence along time or space. This interest also applies to estimates when infection rates are low, like here (Bustamante et al., 2010 *Am J Trop Med Hyg*). It should be noted that infection rates below 1/1000 individuals are usually found in arbovirus surveys in mosquito populations (e.g., Hachiya et al., 2007 *Am J Trop Med Hyg*; Hessson et al., 2015 *Emerg Infect Dis;* Barrera et al., 2019 *Med Vet Entomol*). Thus, we believe that our estimate of the infection rate is of interest to colleagues working on arbovirus epidemiology in the region.

We think, as pointed by the reviewers in several comments, that the description of several aspects of the infection rate estimation was poor. We apologize for this situation. The new version has been extensively modified to improve the description of all information related to the infection rate. First, the Methods section now contains a detailed description of the statistical analysis used to infer the infection rate, including the full dataset and the R script (see answer to comment 4 by reviewer #2 in the Methods section). Secondly, the infection rate is now calculated using the results of the most robust detection method, the isolation in cell culture. This approach avoids overestimation due to a PCR contamination (see answer to comment 2 by reviewer #2 in the Results section). Finally, in the Discussion, we now clearly state the limits of the infection rate estimate and we have eliminated previous claims on the potential impact of the infection rate on public health (see answer to comment 1 by reviewer #1 in the Discussion section). We believe that these modifications have improved the infection rate analysis to a level that makes it of value to other colleagues and worth inclusion in the manuscript.

Reviewer #2: Kaboré and colleagues describe the detection of Sindbis virus (SINV) from a metagenomic survey on mosquitoes from Burkina Faso. This is in itself an interesting result as it is rare to detect arboviruses in mosquitoes with metagenomics.

1/ They show with phylogenetics that their SINV genome belongs to genotype I, which was introduced in Europe from Central Africa. It would be interesting to see a more in-depth phylogeographical analysis to determine if this was a recent introduction from Central Africa.

This comment is related to a previous comment by the same reviewer. We provide below the answer to the previous comment to facilitate review.

The idea of generating a detailed phylogeographical analysis is appealing. We have discussed on this point with experts in phylogeography using Bayesian inference, the method required for a robust analysis of the question (colleagues from Sebastien Duchene’s lab, Pasteur Institute). Their opinion is that our dataset is not adapted to a robust phylogeography analysis because we only have one sequence from Western Africa. For example, the article proposed as example by reviewer #2 generated 19 European sequences and seven African sequences. Together with the sequences in public databases, they included more than 500 sequences to robustly explore virus phylogeography. Hence, for the sake of conciseness, we prefer not to add extra analyses that are not going to improve the phylogenetic analysis already provided in our manuscript.

Given the limits in our sequence dataset, our previous version already described the role of West Africa as the source of European SINV strains as “potential” (line 316 in previous version). Having said that, we acknowledge that other scenarios were not explicitly discussed. For example, West Africa could be a recipient of strains from Central Africa, without being involved in SINV dispersal into Europe. The current version now describes other possible scenarios in the Discussion (lines 332-336; the new text: “Thus, West Africa should also be considered among the potential geographic sources of the European strains. However, other scenarios could have taken place. For example, West Africa could simply be a recipient of strains from Central Africa, without being involved in the spread of SINV to Europe. More sequences from West Africa are needed to address this question through a robust phylodynamic analysis”).

2/ The mosquito infection rate estimation based on the qPCR results is rather weak as a result of concerns of contamination and a lack of transparency in the used methodology.

The new version has been extensively modified to improve the description of all information related to the infection rate. First, the Methods section now contains a detailed description of the statistical analysis used to infer the infection rate, including the full dataset and the R script (see answer to comment 4 by reviewer #2 in the Methods section). Secondly, the infection rate is now calculated using the results of the most robust detection method, the isolation in cell culture. This approach avoids overestimation due to a PCR contamination (see answer to comment 2 by reviewer #2 in the Results section). Finally, in the Discussion, we now clearly state the limits of the infection rate estimate and we have eliminated previous claims on the potential impact of the infection rate on public health (see answer to comment 1 by reviewer #1 in the Discussion section). We believe that these modifications have improved the infection rate analysis to a level that makes it of value to other colleagues and worth inclusion in the manuscript.

3/ In addition, a data availibility statement is lacking with the GenBank accession number for their SINV (it's present in Figure 2), the R code and underlying data for the infection rate calculation, and an SRA accession number for the raw reads for the SINV positive library.

The GenBank accession number of our SINV sequence was already provided in the Results section (line 257 in the previous version). The dataset and the R code for calculation of the infection rate are now provided in Supplementary information 1. The BioProject accession, with the SRA data, is now provided in the manuscript (lines 260-261).

--------------------
